# Evaluation of lexical clarification by patients reading their clinical notes: a quasi-experimental interview study

**DOI:** 10.1186/s12911-020-01286-9

**Published:** 2020-12-15

**Authors:** Hugo J. T. van Mens, Mirte M. van Eysden, Remko Nienhuis, Johannes J. M. van Delden, Nicolette F. de Keizer, Ronald Cornet

**Affiliations:** 1 Department of Medical Informatics, Amsterdam Public Health, Amsterdam UMC, University of Amsterdam, Amsterdam, Netherlands; 2 Department of Research and Development, ChipSoft B.V., Amsterdam, Netherlands; 3 Department of Medical Humanities, Julius Center, University Medical Center, Utrecht, Netherlands

**Keywords:** Consumer health vocabulary, Health literacy, Open notes, Patient access to records, Patient-friendly terminology, Personal health records, Terminology

## Abstract

**Background:**

Patients benefit from access to their medical records. However, clinical notes and letters are often difficult to comprehend for most lay people. Therefore, functionality was implemented in the patient portal of a Dutch university medical centre (UMC) to clarify medical terms in free-text data. The clarifications consisted of synonyms and definitions from a Dutch medical terminology system. We aimed to evaluate to what extent these lexical clarifications match the information needs of the patients. Secondarily, we evaluated how the clarifications and the functionality could be improved.

**Methods:**

We invited participants from the patient panel of the UMC to read their own clinical notes. They marked terms they found difficult and rated the ease of these terms. After the functionality was activated, participants rated the clarifications provided by the functionality, and the functionality itself regarding ease and usefulness. Ratings were on a scale from 0 (very difficult) to 100 (very easy). We calculated the median number of terms not understood per participant, the number of terms with a clarification, the overlap between these numbers (coverage), and the precision and recall.

**Results:**

We included 15 participants from the patient panel. They marked a median of 21 (IQR 19.5–31) terms as difficult in their text files, while only a median of 2 (IQR 1–4) of these terms were clarified by the functionality. The median precision was 6.5% (IQR 2.3–14.25%) and the median recall 8.3% (IQR 4.7–13.5%) per participant. However, participants rated the functionality with median ease of 98 (IQR 93.5–99) and a median usefulness of 79 (IQR 52.5–97). Participants found that many easy terms were unnecessarily clarified, that some clarifications were difficult, and that some clarifications contained mistakes.

**Conclusions:**

Patients found the functionality easy to use and useful. However, in its current form it only helped patients to understand few terms they did not understand, patients found some clarifications to be difficult, and some to be incorrect. This shows that lexical clarification is feasible even when limited terms are available, but needs further development to fully use its potential.

## Background

Patient access to electronic health records (EHRs) is facilitated by patient portals and personal health records. Patients benefit from reading their clinical notes, as it helps them to remember more from what was discussed during consultations and supports them to take care of themselves [1–4]. However, medical data and jargon are difficult to comprehend for most people without a medical background [5–9]. Previous research on lexical simplification (replacing difficult terms with easier terms) and lexical clarification (providing synonyms and definitions to terms) has shown that minimising medical jargon and providing clarifications in medical records may increase comprehension [10–13]. Lexical clarification works similar to infobuttons that are inserted into the EHR to provide additional information [14, 15]. However, rather than retrieving external information resources to aid decision-making, our work is aimed at patients, clarifying medical terminology with a short textual explanation or definition. Therefore, the Dutch university medical centre UMC Utrecht developed functionality in its patient portal to help clarify medical concepts in free-text data sources, such as discharge letters. The functionality used synonyms and definitions from a Dutch thesaurus for care and wellbeing (in Dutch: “Thesaurus Zorg & Welzijn”). Nonetheless, this thesaurus has not been tailored yet to low literacy levels and is not developed to clarify medical concepts to laymen. Previously the terminology had been used as a thesaurus for search functionalities on healthcare websites. Previous research has evaluated what difficulties patients experience when reading their medical records [8, 9], but has not assessed functionality that provides clarifications of difficult terms to patients personal medical records.

We aimed to evaluate to what extent the lexical clarifications match the information needs of patients. First, we assessed whether the right terms were explained, i.e. terms that patients considered difficult. Second, we evaluated whether the terms were explained in the right way, and third, we evaluated how the clarifications and functionality can be improved.

## Study context

The study was carried out at UMC Utrecht, the Netherlands. The functionality was developed by the university itself and implemented in the hospital-wide patient portal in January 2019. The functionality matches free text with terms and synonyms from the thesaurus by text matching and provides a preferred synonym with a definition as a clarification for the matched term. Abbreviations were excluded. The functionality underlines terms that could be matched to the thesaurus, which users can click to view a pop-up window with the clarification. The functionality was activated for treatment reports, medical letters, and test results. No formal evaluation had yet been carried out.

## Methods

### Study design

We carried out an exploratory quasi-experimental before and after interview study. Participants were first asked to read their notes without the functionality and then again with the functionality activated.

### Participants

Participants were invited through the patient panel of the hospital, which included 80 patients willing to be contacted for research on diverse topics related to the quality of care. We included a convenience sample of the first fifteen positive respondents for a 1.5-h interview. The participants received reimbursement for their travel expenses and a gift voucher of 20 euro for their participation.

### Study flow

The interviews were carried out in October and November 2019. The test environment and acceptance environment of the patient portal were used for the study, the first without the functionality, the second with the functionality. During the interviews the participants were asked to read free-text notes from their own EHR aloud. We included medical correspondence between clinicians, discharge summaries, and treatment reports less than 1 year old and routinely available in the patient portal. We excluded notes that were addressed to the participant. Test results were excluded as well, because we did not want to potentially confront the participants with unfamiliar test results. We asked the participants to mark the terms not understood or for which they wished to see an explanation during reading, which we denote as “difficult terms” hereafter. Then, the participants rated the ease of these terms. Next, the functionality was activated and we asked the participants to read the letter again. For each clarification, participants were asked about their thoughts on the clarification and how it could be improved, and to rate the ease and usefulness of the clarification. The terms not marked as difficult, that did get a clarification we denominate as “easy terms” throughout the text. Furthermore, we asked the participants to provide feedback on the functionality, and to rate the ease and usefulness of the functionality. Finally, we removed directly identifying data, such as years and names of the patient or clinicians, and stored the letters including the participants’ terms selection and ratings for further analyses.

### Outcome measures

We collected the following background data from the participants: gender, age, education level, treatment duration in the UMC, whether they had work experience in healthcare, and their health literacy using the validated Dutch version of the Set of Brief Screening Questions (SBSQ) [16]. The primary outcome measure was the number of terms that the participants deemed difficult and that were provided with a clarification by the functionality. The secondary outcome measures were the usefulness of clarifications of the difficult terms compared to the easy terms, the ease and usefulness of the clarification functionality, and the feedback the users provided on the clarifications and functionality. Measurements were carried out on a 100 mm visual analogue scale (VAS, from 0 to 100) and collected with background data on paper case report forms.

### Methods for data acquisition and measurement

The pseudonymized notes were stored. Interviews were audio-recorded, transcribed, and pseudonymized. After the interviews the quantitative data were entered into the electronic data-capture system Castor EDC v2019.3.10 (Ciwit B.V., Amsterdam, The Netherlands).

### Methods for data analysis

We reported the numbers and percentages of the participant characteristics. We calculated the precision and recall of the functionality for each participant. Precision in this study context was defined as the number of difficult terms clarified by the functionality divided by the number of clarifications provided. Recall was defined as the number of difficult terms clarified divided by the number of difficult terms. For each participant we calculated the median number of difficult terms, clarifications provided by the functionality, difficult terms clarified, and the VAS score of the ease and usefulness of the terms, of the clarifications, and of the functionality. We calculated the median and interquartile range (IQR) of the medians per participant. Statistical analysis was carried out in R version 3.6.1 (R Foundation for Statistical Computing, Vienna, Austria) with RStudio 1.2.1335 (RStudio Inc., Boston, MA, USA). The script can be found in Additional file 1.

## Results

### Demographic and other study coverage data

Table 1 lists the characteristics of the fifteen participants. Participants had a median age of 57 (ranging from 34 to 70), eight participants had received higher education, seven were treated at the UMC for more than 10 years, and eight had worked in healthcare in the past. None of the participants had inadequate health literacy. Participants read a median of 2 (IQR 2–3) letters during the interviews, with a median of 214 (IQR 144–395) words per letter, including fourteen outpatient clinic letters, two discharge summaries, and sixteen treatment reports. The letters covered a wide range of medical specialties: angiology, cardiology, dermatology, dietetics, endocrinology, gynaecology, infectiology, nephrology, neurology, nursing, oncology, ophthalmology, physiatry, pulmonology, surgery, and urology. More detailed data can be found in Additional file 2.

**Table 1:** Participant characteristics with the statistic, category, number n and percentage

Statistic	Category	n (%)
Gender	Male	8 (53)
	Female	7 (47)
	Other	0 (0)
Age group	0–18 years	0 (0)
	19–29 years	0 (0)
	30–39 years	1 (7)
	40–49 years	0 (0)
	50–59 years	8 (53)
	60–69 years	4 (27)
	70–79 years	2 (13)
	≥ 80	0 (0)
Education	No education	0 (0)
	Elementary school	0 (0)
	Lower secondary education	2 (13)
	Preparatory vocational secondary education	2 (13)
	Vocational education and training	3 (20)
	Senior general or university preparatory secondary education	0 (0)
	Higher professional education	7 (47)
	Research-oriented higher education	1 (7)
Treatment duration	< 3 years	3 (20)
	3–10 years	5 (33)
	> 10 years	7 (47)
Works in healthcare	Yes, currently	0 (0)
	Yes, in the past	8 (53)
	Never	7 (47)
Health literacy	Inadequate (SBSQ ≤ 2)	0 (0)
	Adequate (SBSQ > 2)	15 (100)

### Study findings and outcome data

**Fig. 1:**
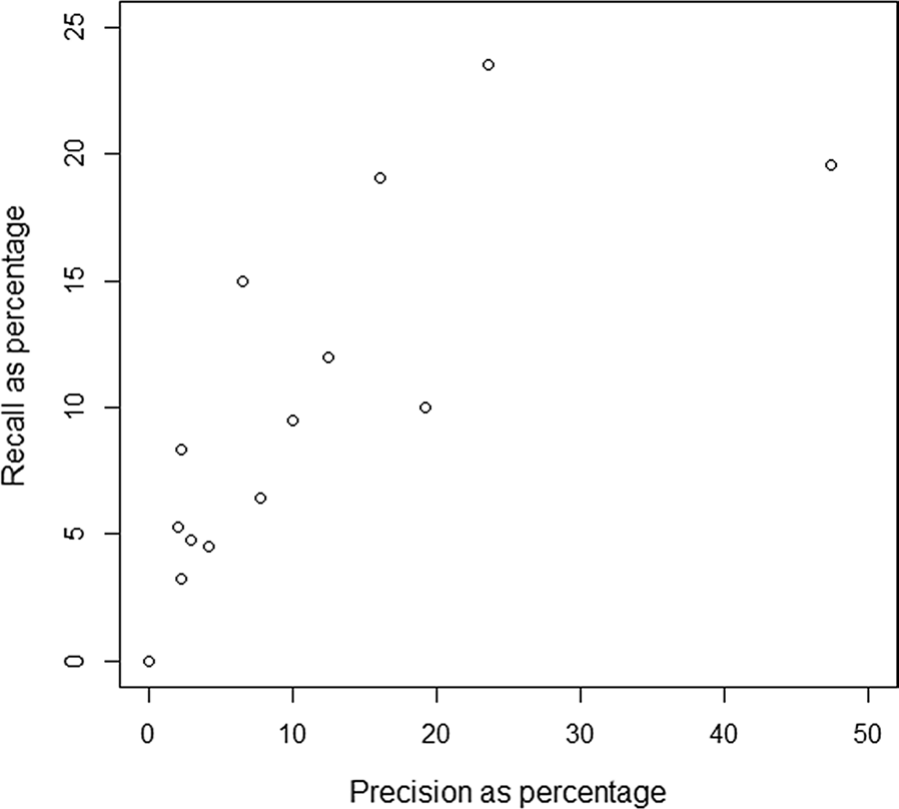
Precision and recall per participant. Two participants had zero difficult terms that were clarified and thus the precision and recall was zero in these cases

Participants marked a median of 21 (IQR 19.5–31) terms in their notes as difficult during the interviews. The functionality provided clarifications for a median of 26 (IQR 22–44) terms per participant, and a median of 2 (IQR 1–4) of these clarifications was provided to terms that participants had also marked as difficult. The median precision per participant was 6.5% (IQR 2.3–14.25%) and the median recall per participant 8.3% (IQR 4.7–13.5%). Two participants did not find any of the terms they deemed difficult clarified. See Fig. 1.

**Fig. 2:**
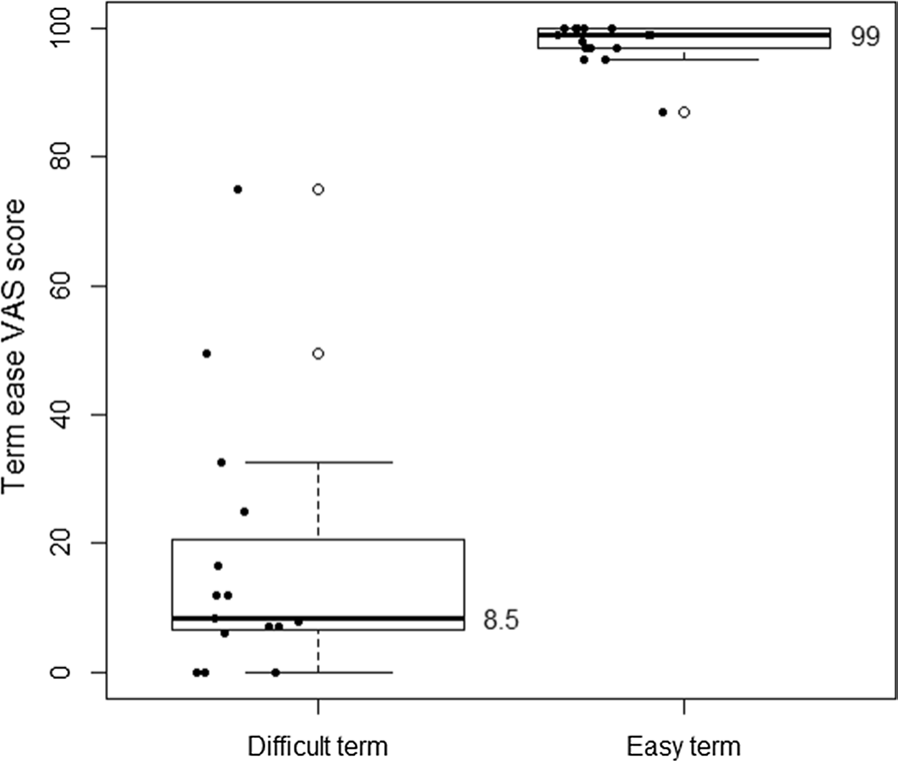
Median ease (from very difficult to very easy) per participant of difficult terms compared to easy terms

**Fig. 3:**
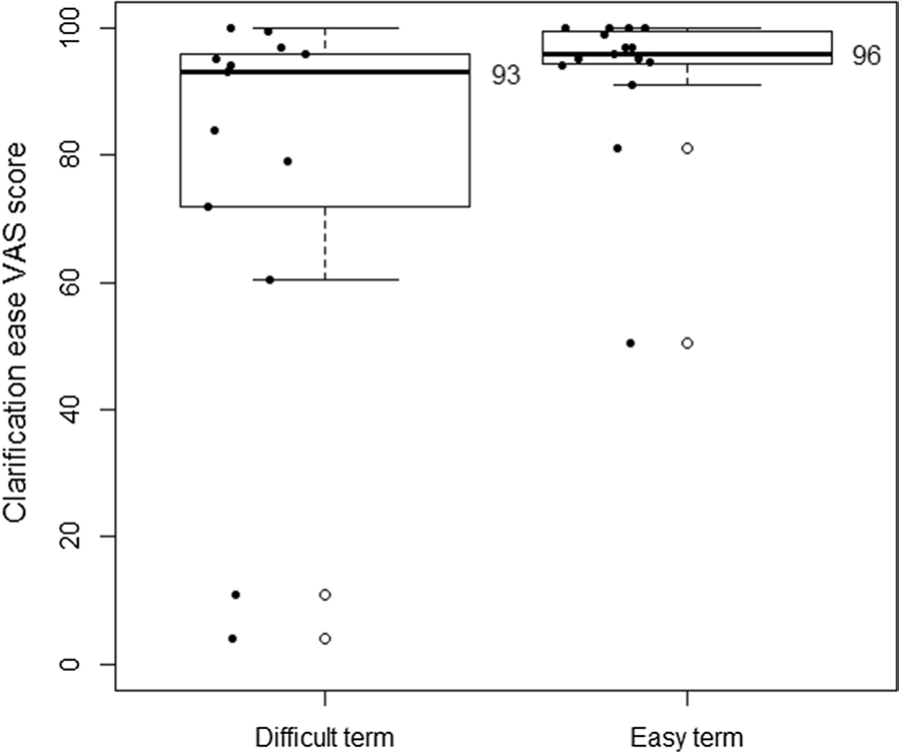
Median ease per participant (from very difficult to very easy) of clarifications of difficult terms compared to clarifications of easy terms

**Fig. 4:**
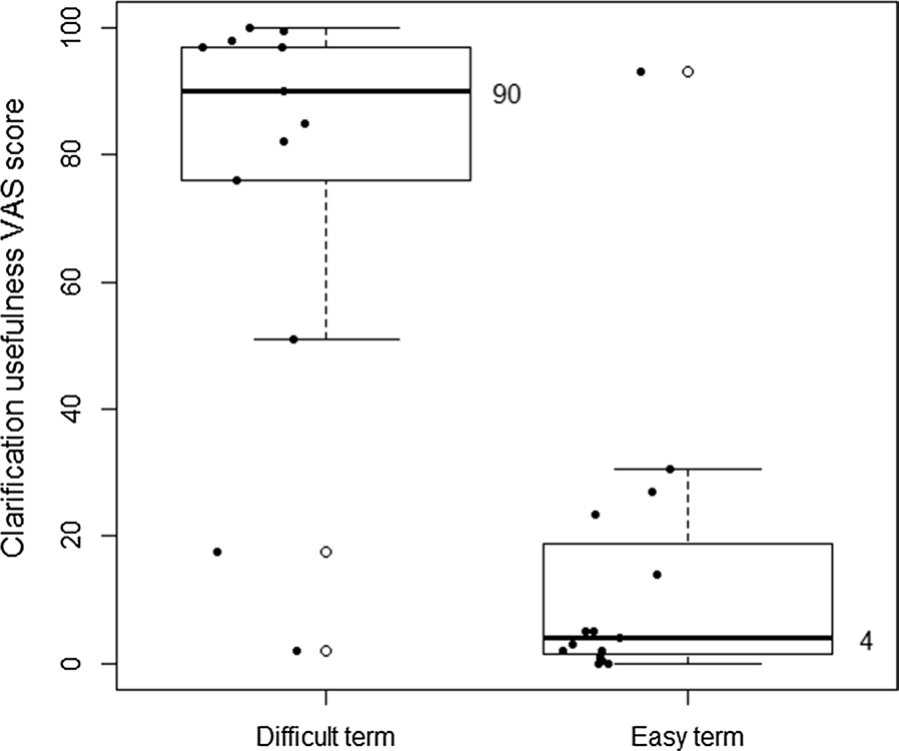
Median usefulness (from not useful at all to very useful) of clarifications of difficult terms compared to clarifications of easy terms

**Fig. 5:**
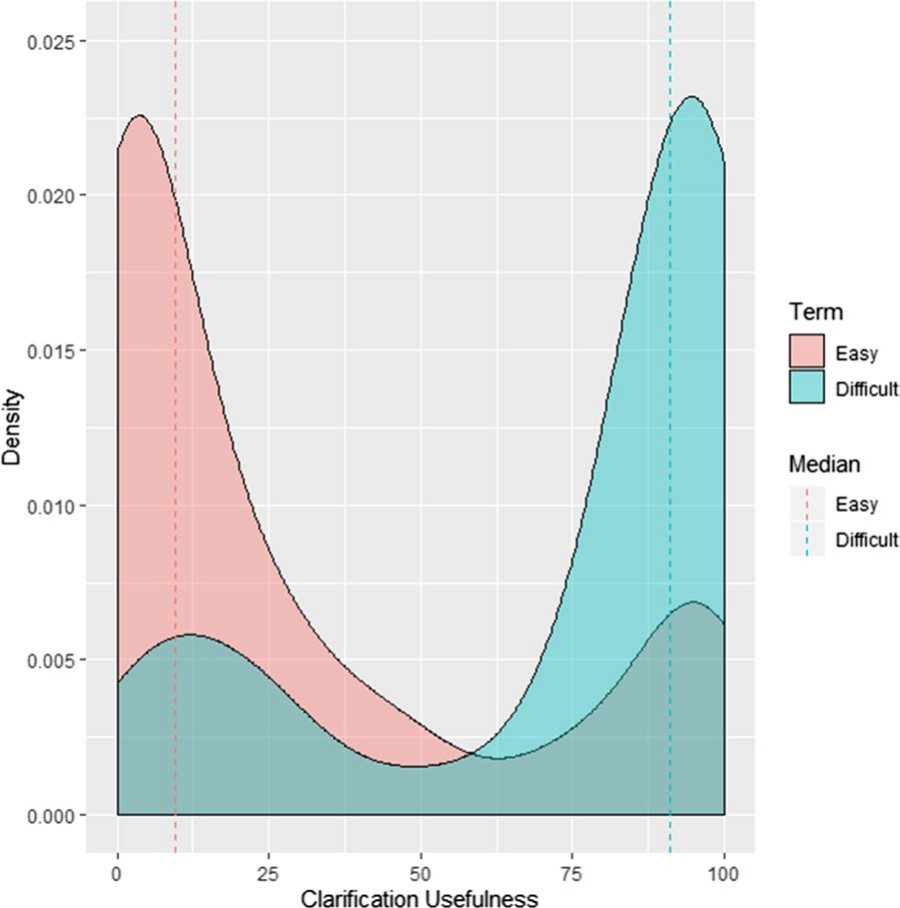
Density plot of usefulness (from not useful at all to very useful) ratings for all terms

Participants rated difficult terms with a median ease of 8.5 (IQR 6.5–20.75), from 0, very difficult, to 100, very easy, and easy terms with a median ease of 99 (IQR 97–100). Difficult term clarifications were rated a median ease of 93 (IQR 72–96) and easy term clarifications 96 (IQR 94.25–99.5). See Figs. 2 and 3. Difficult term clarifications were rated with a median usefulness per participant of 90 (IQR 76–97), from 0, not useful at all, to 100, very useful, while clarifications of easy terms were rated with a usefulness of 4 (IQR 1.5–18.75). See Fig. 4. The density plot in Fig. 5 shows the distribution of overall ratings of the usefulness of clarifications. Participants mostly rated clarifications of easy terms as not useful, because they were not necessary for them personally. However, in some cases, participants thought the clarification was useful somehow anyway because it provided new information and the participants could provide feedback on the clarifications of terms they already knew. They rated difficult term clarifications as useful when it helped them understand the term, but not when the clarification itself was too difficult or incorrect. For example, in a cardiological context, the plaque of blood vessels was clarified with dental plaque. Examples of difficult terms, the most common terms that were clarified, and errors in clarifications are listed in Tables 2, 3, and 4 respectively. For detailed data, see Additional file 2 and Additional file 3. For translations of the examples from Dutch, see Additional file 4.

**Table 2:** Terms marked as difficult by two or more participants, with n as the number of participants that encountered the term

Difficult term	n
eGFR (CKD-EPI)	3
CNS	2
Endocrinology	2
HNP	2
Immune serology	2
Proximal	2
RR	2

**Table 3:** Most common terms clarified by the functionality, with n as the number of participants that encountered the term

Clarification	n
Outpatient clinic	13
Anamnesis	9
Medicine	8
Endocrinology	7
Physical examination	7

**Table 4:** Examples of problems found with some clarifications

Problem with clarification	Example term	Clarification provided to example
Unnecessary	Belly	Belly Part of the trunk between the midriff and the pelvis
Too difficult	Intoxication	Poisoning Distortion of the life functions by a too high concentration of a certain substance in the body
Circular	Neurologists	Neurologists Medical specialists who are specialized in neurology
Homonym (context was about plaque in blood vessels)	Plaque	Plaque White, sticky substance on the teeth and molars in which may occur living and dead bacteria, released tissue cells and food scraps
Related term	Peristaltic	Digestive system Process by which food taken in by the mouth can be made ready for absorption in the blood and the residual products are excreted and the food is then digested

Most participants found the functionality easy to use, with a median ease of 98 (IQR 93.5–99). Two outliers found the functionality not easy or difficult (scores 40 and 50). One of these participants commented it was not clear that the terms were underlined at first and the other found they had to scroll as the clarifications sometimes appeared outside of the window. We observed both issues with other participants as well. The majority of participants found the functionality to be useful, with a median of 79 (IQR 52.5–97), even though participants reported that most clarifications were not useful and the coverage was very low. In general, participants commented that the functionality was fast, easy, inviting to click, well-designed, added value, and liked that it allowed you to do something with the notes, and that one could choose to click or not. They did not like that misspelled words were not taken into account and found a lack of consistency, experienced anxiety, would not use the functionality, thought too many words were underlined, or did not like the design. Participants suggested to add links to further information on the UMC website, enable asking questions, make clarifications more personalized, make the colour of the underlining clearer, and to add more clarifications.

## Discussion

The functionality demonstrated a low precision and recall, which indicates that it does not match the information needs of the patients. However, the patients found the clarifications of the terms they considered difficult to be useful, with some reservations for incorrect and difficult clarifications. Overall, most patients considered the functionality to be easy to use and useful. We observed variance among patients in precision, recall, ease, and usefulness.

The patients were not fully representative for patient portal users in general, as they were actively involved in the patient panel, half had worked in healthcare before, and none of them had inadequate health literacy. We expect the precision and recall to be higher for patients with lower health literacy, and for patients who are still unfamiliar with the topic of their disease and treatment. However, the actively engaged patients from the sample were relatively more knowledgeable about their own health status, and were hence more critical about the functionality. Therefore, the patients were already familiar with many of the terms the functionality clarified, that other persons might not have known, and could provide feedback for improvement from their personal experience and knowledge.

Provider notes are among the most difficult sections of medical records [9]. We have not measured whether the functionality improved the comprehension of patients, but this first requires a further increase of the recall and quality of the clarifications. A strong point of our study is that we read medical correspondence from personal EHRs of the patients. Earlier studies did not use the records from patients themselves [11–13] and have not reported the precision and recall of the functionality that was evaluated. It can be expected that they had a similarly low performance that varied among different patients and notes, and that the increase in comprehension might be lower, when these studies would have used the actual records from patients themselves.

The variance observed between patients is due to multiple factors. On the one hand this includes the (health) literacy of the patient, and his or her familiarity with medical terminology. On the other hand, this might vary according to the medical specialty, writing style of the clinician, and type of free-text source (i.e. treatment reports or medical letters). Further research should address how clarifications can be tailored to the literacy of patients, and how different types of free-text sources can be improved. For example, parts of the free text originate from coded and structured fields in the medical record, such as lab tests and diagnoses, but have lost their underlying coding by being converted to text. It will be easier to clarify the coded data rather than free text, because it is less ambiguous. The difficulty of some clarifications and feedback provided by the patients indicates that the definitions from the thesaurus have a high level of reading difficulty. We thus recommend to make the definitions easier to read. Rather than providing definitions that unambiguously define concepts like dictionaries do, a terminology for lexical clarification should provide explanations of the terms that clarify the meaning to the reader in an appropriate reading level [13]. Further research should therefore address tailoring the definitions to patients’ health literacy levels. Additionally, evaluation studies on lexical clarification functionality should assess the precision and recall of their solutions for different users.

Our results show that in spite of the low recall patients found the clarifications and functionality useful. This is promising for smaller languages where little content for consumer health vocabularies is available. It indicates that it is possible to develop functionality for lexical clarification, starting with a small set of terms and basic text-matching functionality, and to improve it gradually. The results were reported to the developers of the thesaurus and the functionality and will be used for further improvement. This process needs to address the trade-off between introducing more clarifications and having less unnecessary clarifications. More clarifications might increase the recall and usefulness, but will also decrease the precision and may increase the number of incorrect clarifications. For example, many of the unknown terms were abbreviations, which are difficult to disambiguate, even for clinicians. More advanced techniques from natural language processing are required in order to resolve these challenges, that take the context and semantics into account.

## Conclusion

The lexical clarification functionality helped patients to understand terms they did not understand, although the coverage was low. Patients found some clarifications to be difficult or incorrect. Despite low coverage and some problems with available clarifications, patients still found the functionality easy to use and useful. This shows that lexical clarification is feasible and of added value even with limited terminology and coverage. However, incorrect clarifications should be limited to prevent confusion and anxiety.

## Supplementary information

**Additional file 1.** R-script used for the analysis.

**Additional file 2.** Spreadsheet with detailed statistics from R-script output.

**Additional file 3.** Additional figures with term and clarification ease and usefulness on term level and clarification ease and usefulness per participant.

**Additional file 4.** Additional tables with the original Dutch and translated examples of terms marked difficult (Table 2 in the manuscript), most common terms clarified (Table 3 in the manuscript) and problems found (Table 4 in the manuscript).

## Data Availability

The aggregate data are available in the attachments of the manuscript. The Dutch transcripts of the interviews, medical letters, paper case report forms, and castor data are available from the corresponding author on reasonable request. The Dutch interview guide can be obtained from https://purl.org/hjtvanmens/lexicalclarification/interviewguide.

## Abbreviations

HER: Electronic health record
IQR: Interquartile range
SBSQ: Set of Brief Screening Questions
UMC: University medical center
VAS: Visual analogue scale
