# Supplementary material for: Evaluation of lexical clarification by patients reading their clinical notes: a quasi-experimental interview study
Source: BMC Med Inform Decis Mak. 2020 Dec 15;20(Suppl 10):278. doi: 10.1186/s12911-020-01286-9 (PMC7737248; doi:10.1186/s12911-020-01286-9)
Supplement: Supplementary file 3 — Additional file 3. Additional figures with term and clarification ease and usefulness on term level and clarification ease and usefulness per participant. [file 12911_2020_1286_MOESM3_ESM.docx]

Additional file 3: Additional figures

### Term and clarification ease and usefulness on term level


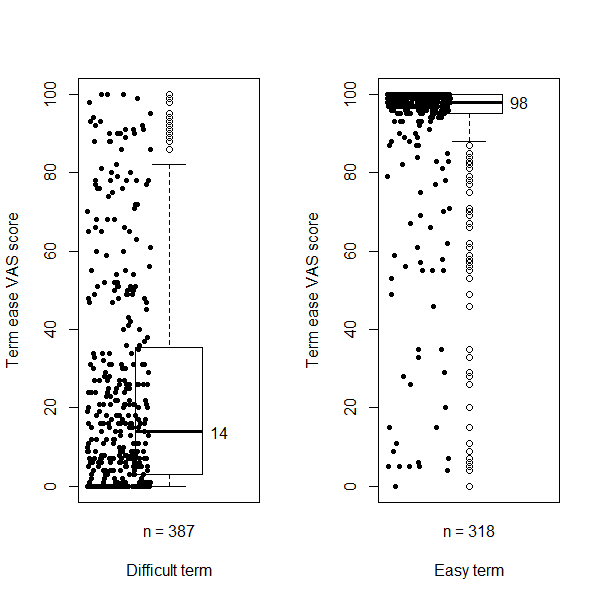


Figure 6 Term ease of difficult terms compared to terms not found difficult (Easy term).


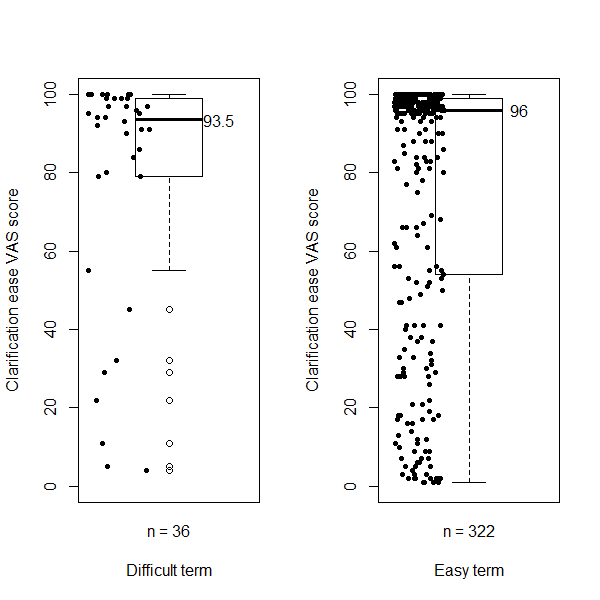


Figure 7 Clarification ease of terms found difficult compared to terms not found difficult.


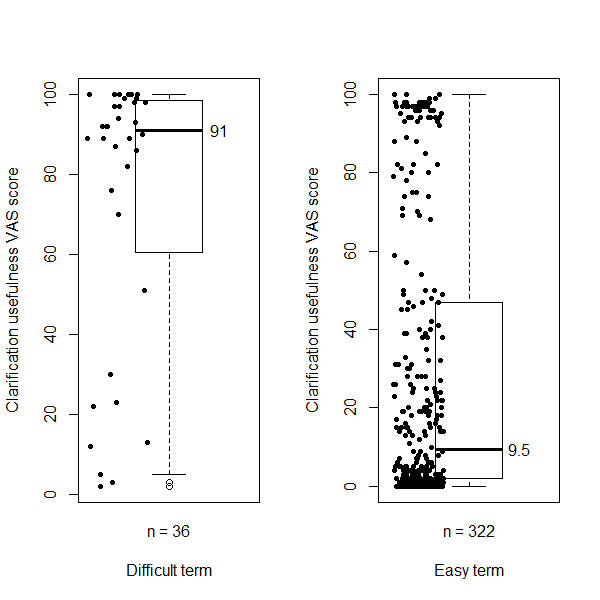


Figure 8 Clarification usefulness of terms found difficult (Difficult term) compared to not found difficult (Easy term)

### Clarification ease and usefulness boxplots per patient


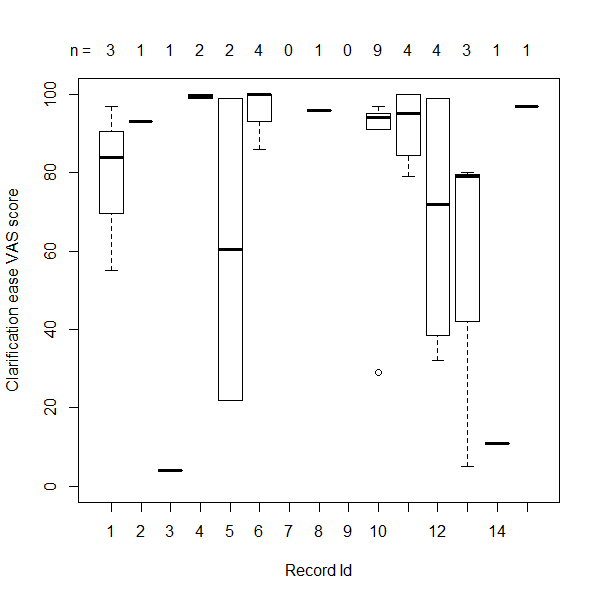


Figure 9 Clarification ease of terms found difficult per participant.


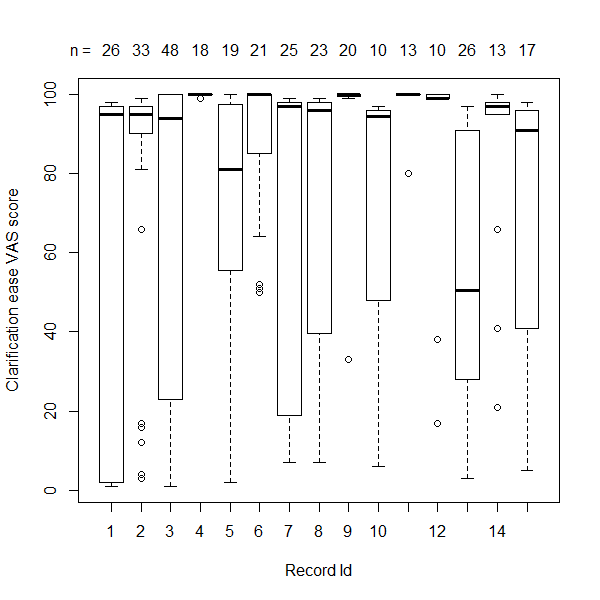


Figure 10 Clarification ease of terms not found difficult per participant.


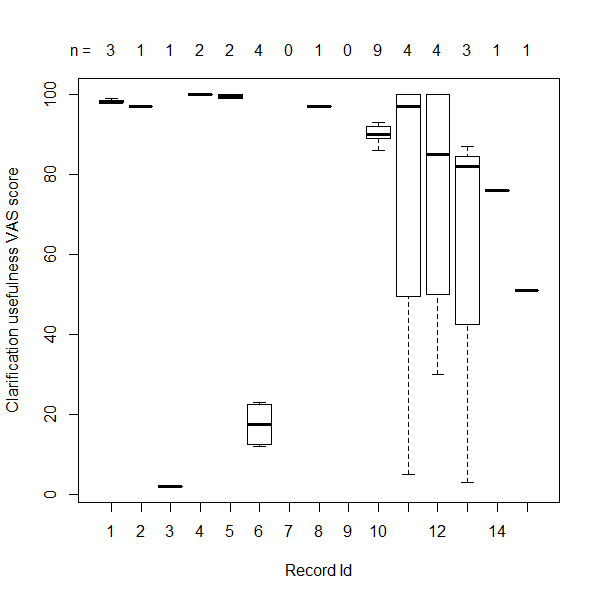


Figure 11 Clarification usefulness of terms found difficult per participant.


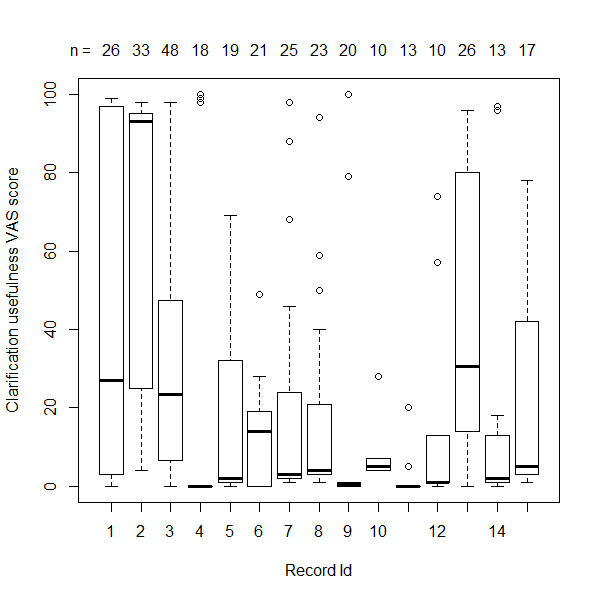


Figure 12 Clarification usefulness of terms not found difficult per participant.
