# Supplementary material for: Evaluation of lexical clarification by patients reading their clinical notes: a quasi-experimental interview study
Source: BMC Med Inform Decis Mak. 2020 Dec 15;20(Suppl 10):278. doi: 10.1186/s12911-020-01286-9 (PMC7737248; doi:10.1186/s12911-020-01286-9)
Supplement: Supplementary file 4 — Additional file 4. Additional tables with the original Dutch and translated examples of terms marked difficult (Table 2 in the manuscript), most common terms clarified (Table 3 in the manuscript) and problems found (Table 4 in the manuscript). [file 12911_2020_1286_MOESM4_ESM.docx]

Additional file 4: Examples

Table 1 Terms marked as difficult by two or more participants with original Dutch terms

| **Difficult term English** | **Difficult term Dutch** | **n** |
| --- | --- | --- |
| eGFR (CKD-EPI) | eGFR (CKD-EPI) | 3 |
| CNS | CNS | 2 |
| Endocrinology | Endocrinologie | 2 |
| HNP | HNP | 2 |
| Immune serology | Immuunserologie | 2 |
| Proximal | Proximaal | 2 |
| RR | RR | 2 |

Table 2 Most common terms clarified with original Dutch terms

| **English** | **Dutch** |
| --- | --- |
| Outpatient clinic | Polikliniek |
| Anamnesis | Anamnese |
| Medicine | Medicijnen |
| Endocrinology | Endocrinologie |
| Physical examination | Lichamelijk onderzoek |

Table 3 Examples of problems found with some clarifications with original Dutch terms and clarifications

| **English term** | **Dutch term** | **English clarification** | **Dutch clarification** |
| --- | --- | --- | --- |
| Belly | Buik | **Belly**  Part of the trunk between the midriff and the pelvis | **Buik**  Deel van romp tussen middenrif en bekken |
| Intoxication | Intoxicatie | **Poisoning**  Distortion of the life functions by a too high concentration of a certain substance in the body | **Vergiftiging**  Verstoring van de levensfuncties door een te hoge concentratie van een bepaalde stof in het lichaam |
| Neurologists | Neurologen | **Neurologists**  Medical specialists who are specialized in neurology | **Neurologen**  Medisch specialisten die gespecialiseerd zijn in de neurologie |
| Plaque | Plaque | **Plaque**  White, sticky substance on the teeth and molars in which may occur living and dead bacteria, released tissue cells and food scraps | **Plaque**  Wittige, kleverige substantie op de tanden en kiezen waarin levende en dode bacteriën, losgelaten weefselcellen en voedselresten voorkomen |
| Peristaltic | Peristalstiek | **Digestive system**  Process by which food taken in by the mouth can be made ready for absorption in the blood and the residual products are excreted and the food is then digested | **Spijsvertering**  Proces waarmee door de mond opgenomen voedsel geschikt wordt gemaakt voor opname in het bloed en de restproducten worden uitgescheiden- het voedsel is dan verteerd |
